# Patient clusters based on HbA1c trajectories: A step toward individualized medicine in type 2 diabetes

**DOI:** 10.1371/journal.pone.0207096

**Published:** 2018-11-14

**Authors:** Tomas Karpati, Maya Leventer-Roberts, Becca Feldman, Chandra Cohen-Stavi, Itamar Raz, Ran Balicer

**Affiliations:** 1 Clalit Research Institute, Tel Aviv, Israel; 2 Israeli National Council of Diabetes, Jerusalem, Israel; International University of Health and Welfare, School of Medicine, JAPAN

## Abstract

**Aims:**

To identify clinically meaningful clusters of patients with similar glycated hemoglobin (HbA1c) trajectories among patients with type 2 diabetes.

**Methods:**

A retrospective cohort study using unsupervised machine learning clustering methodologies to determine clusters of patients with similar longitudinal HbA1c trajectories. Stability of these clusters was assessed and supervised random forest analysis verified the clusters’ reproducibility. Clinical relevance of the clusters was assessed through multivariable analysis, comparing differences in risk for a composite outcome (macrovascular and microvascular outcomes, hypoglycemic events, and all-cause mortality) at HbA1c thresholds for each cluster.

**Results:**

Among 60,423 patients, three clusters of HbA1c trajectories were generated: stable (n = 45,679), descending (n = 6,084), and ascending (n = 8,660) trends, which were reproduced with 99.8% accuracy using a random forest model. In the clinical relevance assessment, HbA1c levels demonstrated a J-shape association with the risk for outcomes. HbA1c level thresholds for minimizing outcomes’ risk differed by cluster: 6.0–6.4% for the stable cluster, <8.0% for the descending cluster, and <9.0 for the ascending cluster.

**Conclusions:**

By applying unsupervised machine learning to longitudinal HbA1c trajectories, we have identified clusters of patients who have distinct risk for diabetes-related complications. These clusters can be the basis for developing individualized models to personalize glycemic targets.

## Introduction

Intensive glycated hemoglobin (HbA1c) control is not always recommended given the inconsistent evidence that lowering HbA1c levels may be associated with increased risk for mortality and other type 2 diabetes-related outcomes [1–6]. Recent guidelines from the American Diabetes Association and the European Association for the Study of Diabetes suggest creating individualized targets to account for the heterogeneity within the population of people with type 2 diabetes [7,8].While the variability in glycemic values over time has been shown to be an important independent risk factor for mortality [9], cardiovascular complications [10], and cognitive performance [11], current guidelines do not address how to incorporate glycemic level trajectories when characterizing individual risk.

Creating sub-groups of this complex population may help to identify underlying common characteristics for improving the appropriateness of treatment goals. This is supported by a growing body of evidence showing that stratification of a population into more homogenous sub-groups can achieve better prediction of individualized models [12,13]. There are multiple methodologies to do so, including clustering using laboratory data trajectories with demonstrated utility in differentiating stages of chronic diseases [14,15]. Machine learning algorithms have also been used to discover hidden patterns in complex datasets through unsupervised methodologies, which can yield clusters of individuals with similar behaviors or characteristics. These techniques can be valuable in identifying groups of patients with type 2 diabetes who have distinct risk profiles that are different from previous findings.

As part of a larger study to create a tool that provides individualized HbA1c targets for optimal long-term type 2 diabetes risk management, this sub-study aimed to: (1) characterize clusters of similar patients based on HbA1c trajectories over three years and (2) evaluate the clinical relevance of these clusters by assessing the associated risk for type 2 diabetes outcomes for each cluster.

## Methods

### Setting and data source

All-cause mortality, demographic, clinical, and laboratory data were obtained from the Clalit Health Services (Clalit) healthcare data warehouse. Clalit is the largest of the four payer/provider health funds in Israel, providing healthcare services to over four million patients, approximately 53% of the total Israeli population. This study was performed using deidentified electronic health record (EHR) data from Clalit’s fully integrated database, which centralizes data from community clinics, hospital visits, laboratory tests and results, and medication dispensing. This study was approved by Clalit’s internal ethics review board.

### Study design

**Fig 1:**
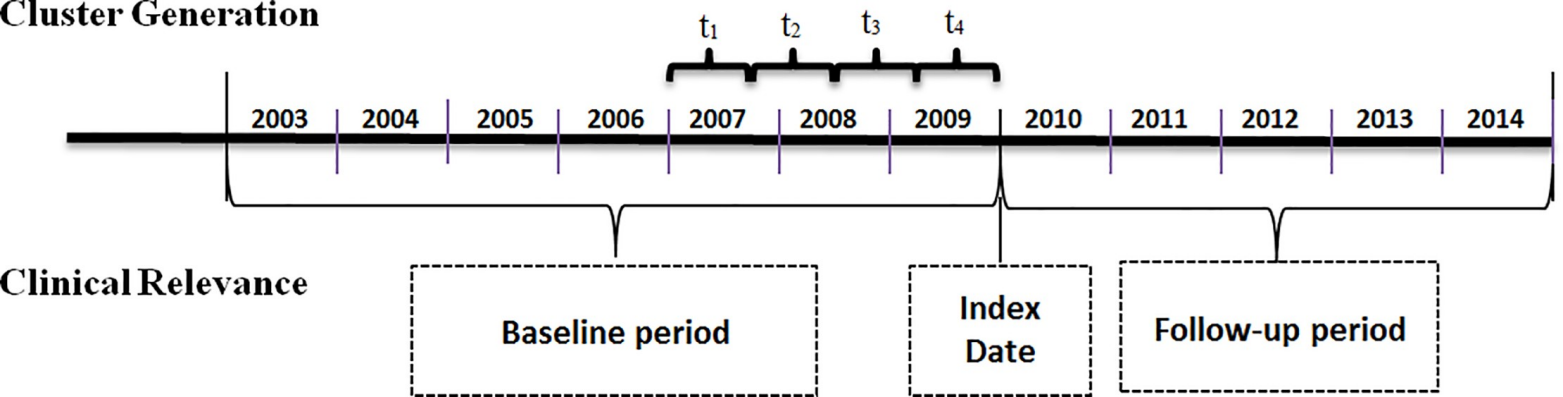
Study design. Abbreviations: t_1,_ t_2,_ t_3_ and t_4_ are the four nine-month periods HbA1c measures used for cluster analysis.

This study is a longitudinal retrospective cohort study among patients with type 2 diabetes having disease duration of three to seven years prior to January 1, 2010 (index date). The first part of this study derived clusters employing unsupervised machine learning techniques using each patient's HbA1c history taken from the three years prior to the index date. The second part, also addressing the first objective, uses a supervised machine learning model to determine the clusters’ reproducibility. Finally, we tested the clinical relevance of the derived clusters by evaluating multivariable five-year risk for type 2 diabetes outcomes with the baseline period from January 1, 2003 through December 31, 2009 and the follow-up period from January 1, 2010 through December 31, 2014 (Fig 1).

### Study population

The study population consists of Clalit members included in the Clalit diabetes registry who are identified via an algorithm previously described in detail [16]. These patients with short to medium duration type 2 diabetes were selected to allow sufficient time for disease-related complications to develop, but not too long to have developed irreversible damage. Additionally, patients included had at least three years of continuous membership at Clalit prior to the index date. Patients were excluded if they had concurrent chronic conditions, such as cancer, chronic infectious disease (AIDS, Hepatitis B/C/Delta), or hepatic cirrhosis.

### Longitudinal HbA1c measures

We assembled HbA1c trajectories based on available and imputed laboratory data, excluding extreme outliers (defined as seven standard deviations from the cohort mean). HbA1c trajectories were aggregated into four time periods of nine months (t1, t2, t3, t4) and the average were used when there were multiple measurements in each time frame. The decision to use nine-month time frames was based on the observation that more than 80% of the study cohort had two consecutive HbA1c tests within nine months.

Only patients who had HbA1c measures in at least three of the four nine-month time periods were included in the unsupervised cluster analysis; otherwise, they were designated as a separate group labeled ‘Undefined Cluster.’ Among patients with HbA1c measures in only three of the four time periods, missing time frame values were imputed using linear models based on the other three HbA1c values. The imputed dataset was randomly divided into a training set (60% of the dataset) and a validation set (40%). Four separate models were created for imputation, one for each nine-month time period (S1 Fig).

### Clusters generation

A longitudinal unsupervised trajectory clustering methodology was implemented using the “*traj” R* package (version 1.2) [17,18]. The methodology includes feature engineering by generating 24 different features derived from HbA1c trajectories (S1 Table). The most relevant of these HbA1c measures were selected using factor analysis. The selected measures were then used for cluster generation. The optimal number of clusters were calculated using the *“NbClust”* algorithm (R package) as described by Charrad et al. [19]. The calculation of the optimal number of clusters was based on the most frequent number recommended by 26 different methods [19]. This is the standard number of methods included in the analysis package, which assumes a voting approach in determining the optimal number of clusters.

This study utilized K-means clustering of the selected features, similar to how Jacob et al. [20] used K-means clustering to uncover patterns in metabolite levels of pregnant patients’ data. The K-means default parameters were defined by the *traj* functions. K-means is an algorithm that characterizes the clusters by searching for the optimal centers of the data points on a multidimensional space, using randomly and iteratively resampled data points until the distance between those centers and the other points in the same cluster is minimized. The stability of the clusters was assessed using fixed point cluster analysis as described by Hennig [21]. A Jaccard similarity index of 0.95 or higher, indicates that the cluster is highly stable [22,23].

### Reproducibility of clusters using a supervised algorithm

One limitation of unsupervised algorithms is that they cannot always be reproduced on new data. In order to support the reproducibility of our findings on any new dataset, a supervised random forest algorithm was developed on a training subset, 60% of the original data, and the resulting algorithm was validated using a test dataset. This algorithm assembles multiple iterations of decision trees to determine how accurately it can yield the predicted clusters that were produced with the unsupervised algorithm. Through discovery of the rules that were used by the unsupervised K-means algorithm, the random forest algorithm classifies the individuals into clusters. The accuracy was assessed as the number of correctly predicted clusters divided by the number of patients in the dataset. The advantage of this kind of algorithm is that it yields very accurate models and prevents overfitting.

### Clinical relevance assessment of clusters

The clinical relevance of the clusters was determined by comparing the five-year risk for type 2 diabetes outcomes at various levels of HbA1c across the cluster groups. This HbA1c was defined as the first test value after the index date (post-index HbA1c) during the follow-up period. Patients without a post-index HbA1c value were excluded from this clinical relevance assessment.

Type 2 diabetes outcomes were defined as a composite outcome of macrovascular and microvascular complications, hypoglycemic events and all-cause mortality, and the first event to occur in the follow-up period indicated an outcome. Macrovascular outcomes were any incident event of one of the following conditions: myocardial infarction (MI), unstable angina pectoris (UAP), coronary artery bypasses graft (CABG), percutaneous transluminal coronary angioplasty (PTCA), and cerebrovascular accident (CVA). Microvascular complications were the first recorded new diagnosis of diabetic retinopathy (DR), diabetic neuropathy (DNeu), diabetic nephropathy (DNeph), a lower extremity ulcer (LEU), or a lower extremity amputation (LEA) (S2 Table). All diagnoses prior to the index date were considered prevalent comorbidities and were not considered outcomes.

In addition to post-index HbA1c, covariates included in our analyses were age (in years), sex, socio-economic status (SES, as low, medium, and high categories), obesity (BMI of 30 kg/m^2^ or higher), smoking status (current smokers, former smokers, non-smokers, and unknown), diagnosis of hypertension, diagnosis of congestive heart failure, history of hypoglycemic episodes, and if chronic disease medications were dispensed (at least one or more dispensed medication of any of the following: cholesterol lowering drugs, agents acting on the renin-angiotensin system, insulin, and hypoglycemic agents). All these variables were collected as of the index date, with the last measure of multiple values used when relevant.

The risk for the composite outcome was assessed at different HbA1c levels for each cluster, grouped according to eight categories: <6.0% (<42 mmol/mol), 6.0–6.4% (42–47 mmol/mol, reference group), 6.5–6.9% (47–53 mmol/mol), 7.0–7.4% (53–58 mmol/mol), 7.5–7.9% (58–64 mmol/mol), 8.0–8.4% (64–69 mmol/mol), 8.5–8.9% (69–75 mmol/mol) and ≥9.0% (≥75 mmol/mol).

### Statistical analysis

For continuous variables, p values where calculated using ANOVA. In case of heteroscedasticity (measured using the Bartlett test), White correction was applied. For categorical variables, Chi square test was used if all the cells were higher than 5. In cases where one cell or more had 5 or less observations, the Kruskal-Wallis test was used. Multivariable logistic regression models were generated by adjusting post-index HbA1c categories for all covariates. All analyses were performed using the R statistical software version 3.2.2 and the previously named packages [24].

## Results

We identified 85,783 patients meeting the inclusion criteria, of which 60,423 patients (70.4%) with 217,133 associated HbA1c valid measures were included in the sample for clustering.

Table 1 shows the main demographic and clinical characteristics of the overall study population and each of the patient clusters. The mean age of the study cohort was 63.6 years, 52.6% of the patients were female, 28.3% had a low SES, and 30.0% had a high SES. The mean post-index HbA1c was 7.5% (58 mmol/mol).

**Table 1:** Demographic and clinical baseline characteristics for the overall study population and for the longitudinal trajectory clusters.

Characteristic		Overall Population	Stable cluster	Descending cluster	Ascending cluster	Undefined Cluster	p-value
		(n = 85,783)	(n = 45,679)	(n = 6,084)	(n = 8,660)	(n = 25,360)	
Age*	Mean (SD)	63.6 (13.4)	66.0 (12.0)	62.0 (12.2)	59.9 (12.0)	61.0 (15.4)	<0.001
						
Sex	Female	45,129 (52.6%)	25,313 (55.4%)	3,061 (50.3%)	4,362 (50.4%)	12,393 (48.9%)	
	Male	40,654 (47.4%)	20,366 (44.6%)	3,023 (49.7%)	4,298 (49.6%)	12,967 (51.1%)	<0.001
SES	Low	24,186 (28.3%)	11,866 (26.0%)	2,257 (37.2%)	2,887 (33.5%)	7,176 (28.4%)	
	Medium	35,678 (41.7%)	19,465 (42.7%)	2,349 (38.7%)	3,525 (40.9%)	10,339 (40.9%)	<0.001
	High	25,673 (30.0%)	14,239 (31.2%)	1,468 (24.2%)	2,213 (25.7%)	7,753 (30.7%)	
	Missing (%)	246 (0.3%)	109 (0.2%)	10 (0.2%)	35 (0.4%)	92 (0.4%)	
HbA1c (%)^†^	Mean (SD)	7.5 (1.7)	7.1 (1.2)	7.8 (1.8)	8.7 (1.9)	7.7 (2.0)	
	Median (IQR)	7.0 (6.4–8.0)	6.8 (6.4–7.5)	7.4 (6.6–8.7)	8.3 (7.3–9.9)	7.0 (6.3–8.6)	<0.001
HbA1c (mmol/mol) ^†^	Mean (SD)	58.3 (18.4)	54.2 (13.4)	62.1 (19.7)	71.9 (21.2)	60.4 (22.2)	
	Median (IQR)	53.0 (46.5–63.9)	50.8 (46.5–58.5)	57.4 (48.6–71.6)	67.2 (56.3–84.7)	53.0 (45.4–70.5)	<0.001
	Missing (%)	3,900 (4.5%)	519 (1.1%)	88 (1.4%)	93 (1.1%)	3,200 (12.6%)	
Diabetes Duration (months)	Mean (SD)	62.4 (14.1)	60.8 (14.1)	65.6 (13.8)	64.0 (14.0)	62.6 (14.1)	
	Median (IQR)	63.0 (50.0–75.0)	60.0 (48.0–73.0)	68.0 (55.0–78.0)	65.0 (52.0–77.0)	63.0 (50.0–75.0)	<0.001
Smoking status	Non smoker	55,112 (65.2%)	30,375 (67.1%)	3,746 (62.0%)	5,290 (61.5%)	15,701 (64.0%)	
	Current smoker	16,020 (19.0%)	7,151 (15.8%)	1,312 (21.7%)	1,913 (22.2%)	5,644 (23.0%)	<0.001
	Past smoker	13,343 (15.8%)	7,770 (17.2%)	981 (16.2%)	1,400 (16.3%)	3,192 (13.0%)	
	Missing (%)	1,308 (1.5%)	383 (0.8%)	45 (0.7%)	57 (0.7%)	823 (3.2%)	
BMI	Mean (SD)	30.3 (5.9)	30.3 (5.7)	30.7 (6.0)	31.4 (6.1)	29.8 (6.0)	<0.001
(kg/m^2^)							
	<18.5	320 (0.4%)	137 (0.3%)	26 (0.4%)	23 (0.3%)	134 (0.6%)	
	18.5–24.9	13,250 (15.8%)	6,907 (15.3%)	857 (14.2%)	1,002 (11.7%)	4,484 (18.8%)	<0.001
	25–29.9	31,346 (37.5%)	17,254 (38.1%)	2,151 (35.7%)	2,878 (33.5%)	9,063 (38.1%)	
	30–34.9	23,488 (28.1%)	12,845 (28.4%)	1,766 (29.3%)	2,608 (30.4%)	6,269 (26.3%)	
	35+	15,236 (18.2%)	8,085 (17.9%)	1,232 (20.4%)	2,076 (24.2%)	3,843 (16.2%)	
	Missing (%)	2,143 (2.5%)	451 (1.0%)	52 (0.9%)	73 (0.8%)	1,567 (6.2%)	
Previous	Macrovascular	8,923 (10.4%)	5,032 (11.0%)	787 (12.9%)	939 (10.8%)	2,165 (8.5%)	<0.001
Comorbidities	Microvascular	18,307 (21.3%)	10,099 (22.1%)	1,930 (31.7%)	2,504 (28.9%)	3,774 (14.9%)	<0.001
	Hypoglycemia	8,019 (9.3%)	3,742 (8.2%)	828 (13.6%)	801 (9.2%)	2,648 (10.4%)	<0.001
	Hypertension	29,828 (34.8%)	16,584 (36.3%)	2,421 (39.8%)	3,299 (38.1%)	7,524 (29.7%)	<0.001
	Non-CKD	65,257 (91.5%)	34,812 (90.0%)	4,586 (91.8%)	6,952 (93.4%)	18,907 (93.4%)	<0.001
CKD							
	CKD 3A	4,009 (5.6%)	2,601 (6.7%)	238 (4.8%)	310 (4.2%)	860 (4.2%)	
	CKD 3B	1,187 (1.7%)	732 (1.9%)	75 (1.5%)	89 (1.2%)	291 (1.4%)	
	CKD 4	270 (0.4%)	158 (0.4%)	27 (0.5%)	32 (0.4%)	53 (0.3%)	
							
	CKD5/RRT	615 (0.9%)	361 (1.0%)	65 (1.3%)	61 (0.9%)	128 (0.7%)	
	Unknown	14,445 (16.8%)	7,015 (15.4%)	1,093 (18.0%)	1,216 (14.0%)	5,121 (20.2%)	
Type 2 diabetes treatment medication class	Insulin (fast-acting)	1,818 (2.1%)	792 (1.7%)	392 (6.4%)	341 (3.9%)	293 (1.2%)	<0.001
	Insulin (non-exclusively fast-acting)	4,147 (4.8%)	1,704 (3.7%)	852 (14.0%)	895 (10.3%)	696 (2.7%)	<0.001
	Biguanides	59,572 (69.4%)	34,479 (75.5%)	5,162 (84.8%)	7,390 (85.3%)	12,541 (49.5%)	<0.001
	Sulfonamides	13,436 (15.7%)	6,582 (14.4%)	1,735 (28.5%)	2,223 (25.7%)	2,896 (11.4%)	<0.001
	Thiazolidines	1,044 (1.2%)	527 (1.2%)	249 (4.1%)	149 (1.7%)	119 (0.5%)	<0.001
	DDP-4 Inhibitors	2,030 (2.4%)	1,133 (2.5%)	198 (3.3%)	420 (4.8%)	279 (1.1%)	<0.001
	Non-Insulin only	58,582 (68.3%)	34,402 (75.3%)	4,583 (75.3%)	6,883 (79.5%)	12,714 (50.1%)	
Type 2 diabetes treatment type	Insulin +/- Non-insulin	4,892 (5.7%)	2,029 (4.4%)	1,014 (16.7%)	1,030 (11.9%)	819 (3.2%)	<0.001
	No treatment	22,309 (26.0%)	9,248 (20.2%)	487 (8.0%)	747 (8.6%)	11,827 (46.6%)	
Other chronic medications	Beta-blockers	22,830 (26.6%)	13,687 (30.0%)	1,602 (26.3%)	2,162 (25.0%)	5,379 (21.2%)	<0.001
	Ca-Blockers	18,348 (21.4%)	11,292 (24.7%)	1,258 (20.7%)	1,596 (18.4%)	4,202 (16.6%)	<0.001
	ACE/ARB	33,663 (39.2%)	19,459 (42.6%)	2,819 (46.3%)	3,722 (43.0%)	7,663 (30.2%)	<0.001
	Statins	36,220 (42.2%)	21,485 (47.0%)	2,805 (46.1%)	3,711 (42.9%)	8,219 (32.4%)	<0.001

Note: Values for continuous variables were presented as mean (SD) and median (IQR). For categorical variables, the n (%) format was used.

Abbreviations: SD, standard deviation; IQR, interquartile range; SES, socioeconomic status; HbA1c, glycated hemoglobin; BMI, body mass index; CKD, chronic kidney disease; RRT, renal replacement therapy; DDP-4 inhibitor, dipeptidyl peptidase-4 inhibitor; Ca-blocker, calcium blocker; ACE, Angiotensin-converting-enzyme inhibitors; ARB, Angiotensin II receptor blockers.

*Age at the date of the index HbA1c measure.

^†^First HbA1c level after the index date (post-index HbA1c).

### Cluster generation

Of the 24 measures generated through feature engineering [17,18], the most relevant measures selected by factor analysis were the change in HbA1c values from t1 to t4, mean of the absolute first differences in HbA1c values, and the ratio of the maximum absolute second difference to mean absolute first difference of HbA1c values. The *NbClust* algorithm indicated that the recommended number considered as the optimal number of clusters was three (S1 Text and S3 Table).

**Fig 2:**
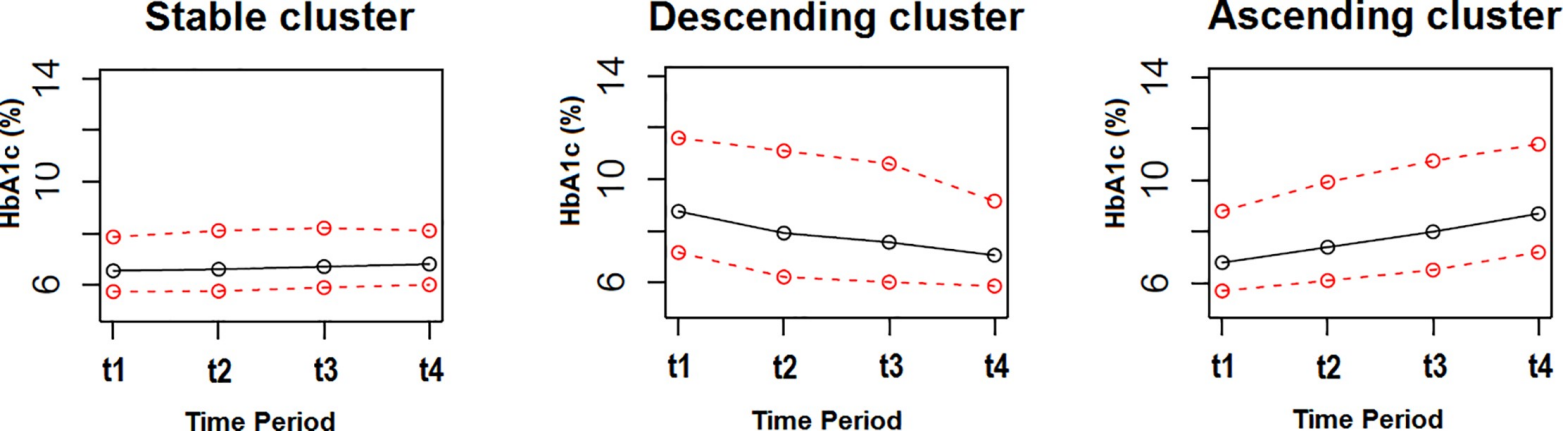
Historical HbA1c trajectories by cluster. Abbreviations: t_1,_ t_2,_ t_3_ and t_4_ are the four nine-month periods HbA1c measures used for cluster analysis. The graphs show the median, 10 and 90 percentiles of HbA1c measures for each time frame of nine months (a total of three years). The stable cluster demonstrated stable levels of HbA1c over time. The ascending and descending clusters demonstrated non-stable levels with an increasing and decreasing trend, respectively.

The distribution of the clusters was as follows: stable cluster: 45,679 patients with a stable HbA1c trend over time; decreasing cluster: 6,084 patients with a descending trend over time; and ascending cluster: 8,660 patients with an ascending trend over time. undefined cluster included 25,360 patients and was also included in the analyses for comparison. The Jaccard similarity indexes for the resulting clusters were 0.99 for the stable cluster, 0.99 for the decreasing cluster, and 0.98 for the ascending cluster. Fig 2 shows the median trajectory (with the lower and upper 10%) for each cluster.

### Reproducibility of the clusters using a supervised algorithm

The random forest model for classifying patients into specific clusters had an accuracy of 99.8% in the test dataset.

### Baseline characteristics by clusters

Table 1 shows a comparison of the baseline characteristics of the study population by clusters. The stable cluster had the oldest population while the ascending cluster had the youngest population (66.0 [12.0] vs. 59.9 [12.0] years old, p<0.001). The stable cluster had a higher proportion of women (55.4%) compared to the other clusters that had approximately 50% women each (p<0.001), and a lower proportion of current smokers (15.8% vs. 21.7%-23.0%, p<0.001). The stable and undefined clusters had the highest proportion of patients with high SES (31.2% and 30.7%, respectively) while the descending cluster had the highest proportion of patients with low SES (37.2%, p<0.01). The stable cluster’s patients had the lowest average index glycemic level (HbA1c: 7.1 [1.2]%, 54.2 [13.4] mmol/mol) while the ascending cluster’s patients had the highest average level (HbA1c: 8.7 [1.9]%, 72 [21.2] mmol/mol, p<0.001). The ascending cluster had the highest proportion of patients being treated only with non-insulin hypoglycemic medications (79.5% in the ascending cluster vs 75.3% for the descending and stable clusters, p<0.001), while the descending cluster had the highest proportion of patients treated with insulin (and a possible additional non-insulin medication) (16.7% vs 11.9% for the ascending cluster and 4.4% for the stable cluster, p<0.001). The stable cluster had 20.2% no treatment compared to the 8.0% in both the descending and ascending clusters, while the undefined cluster had the highest proportion of untreated patients (46.6%).

### Clinical relevance assessment by clusters

The descending and ascending clusters showed the higher proportion of baseline prevalent and incident outcomes for micro and macrovascular complications compared to the stable cluster (p<0.001) (Tables 1 and 2).

**Table 2:** Outcomes for the overall study population and for the longitudinal trajectory clusters.

Characteristic		Overall Population	Stable Cluster	Descending Cluster	Ascending Cluster	Undefined Cluster	p-value
		(n = 85,783)	(n = 45,679)	(n = 6,084)	(n = 8,660)	(n = 25,360)	
Microvascular outcomes ^‡^	DR	4,902 (5.7%)	2,260 (4.9%)	569 (9.4%)	719 (8.3%)	1,354 (5.3%)	<0.001
	DNeu	7,499 (8.7%)	4,128 (9.0%)	634 (10.4%)	1,105 (12.8%)	1,632 (6.4%)	<0.001
	DNeph	5,641 (6.6%)	3,070 (6.7%)	469 (7.7%)	724 (8.4%)	1,378 (5.4%)	<0.001
	LEU	3,582 (4.2%)	1,677 (3.7%)	406 (6.7%)	490 (5.7%)	1,009 (4.0%)	<0.001
	LEA	823 (1.0%)	307 (0.7%)	116 (1.9%)	108 (1.2%)	292 (1.2%)	<0.001
	Microvascular	18,248 (21.3%)	9,687 (21.2%)	1,704 (28.0%)	2,496 (28.8%)	4,361 (17.2%)	<0.001
Hypoglycemic events		9,131 (10.6%)	4,809 (10.5%)	1,070 (17.6%)	1,201 (13.9%)	2,051 (8.1%)	<0.001
Macrovascular outcomes ^‡^	MI	3,720 (4.3%)	1,837 (4.0%)	328 (5.4%)	409 (4.7%)	1,146 (4.5%)	<0.001
	UAP	3,454 (4.0%)	1,801 (3.9%)	263 (4.3%)	430 (5.0%)	960 (3.8%)	<0.001
	CABG	1,431 (1.7%)	713 (1.6%)	126 (2.1%)	164 (1.9%)	428 (1.7%)	0.007
	PTCA	4,658 (5.4%)	2,454 (5.4%)	394 (6.5%)	542 (6.3%)	1,268 (5.0%)	<0.001
	CVA	4,915 (5.7%)	2,570 (5.6%)	437 (7.2%)	523 (6.0%)	1,385 (5.5%)	<0.001
	Macrovascular	12,093 (14.1%)	6,367 (13.9%)	1,000 (16.4%)	1,331 (15.4%)	3,395 (13.4%)	<0.001
All-cause mortality		11,283 (13.2%)	5,640 (12.3%)	930 (15.3%)	951 (11.0%)	3,762 (14.8%)	<0.001

Abbreviations: DR, diabetic retinopathy; DNeu, diabetic neuropathy; DNeph, diabetic nephropathy; LEU, low extremity ulcers; LEA, low extremity amputations; MI, myocardial infarction; UAP, unstable angina pectoris; CABG, coronary artery bypass graft; PTCA, percutaneous transluminal coronary angioplasty; CVA, cerebrovascular disease.

^‡^Outcomes refer to the first incident event occurring after the index HbA1c measure date (follow up period).

Hypoglycemic events were more frequent in patients in the descending cluster in both the baseline and follow-up periods (p<0.001). Mortality was also higher in the descending cluster (15.3% vs 11.0% and 12.3% for the stable and ascending clusters, respectively; p<0.001). The undefined cluster showed relatively low levels of micro and macrovascular complications, but had higher mortality rates (14.8%).

**Fig 3:**
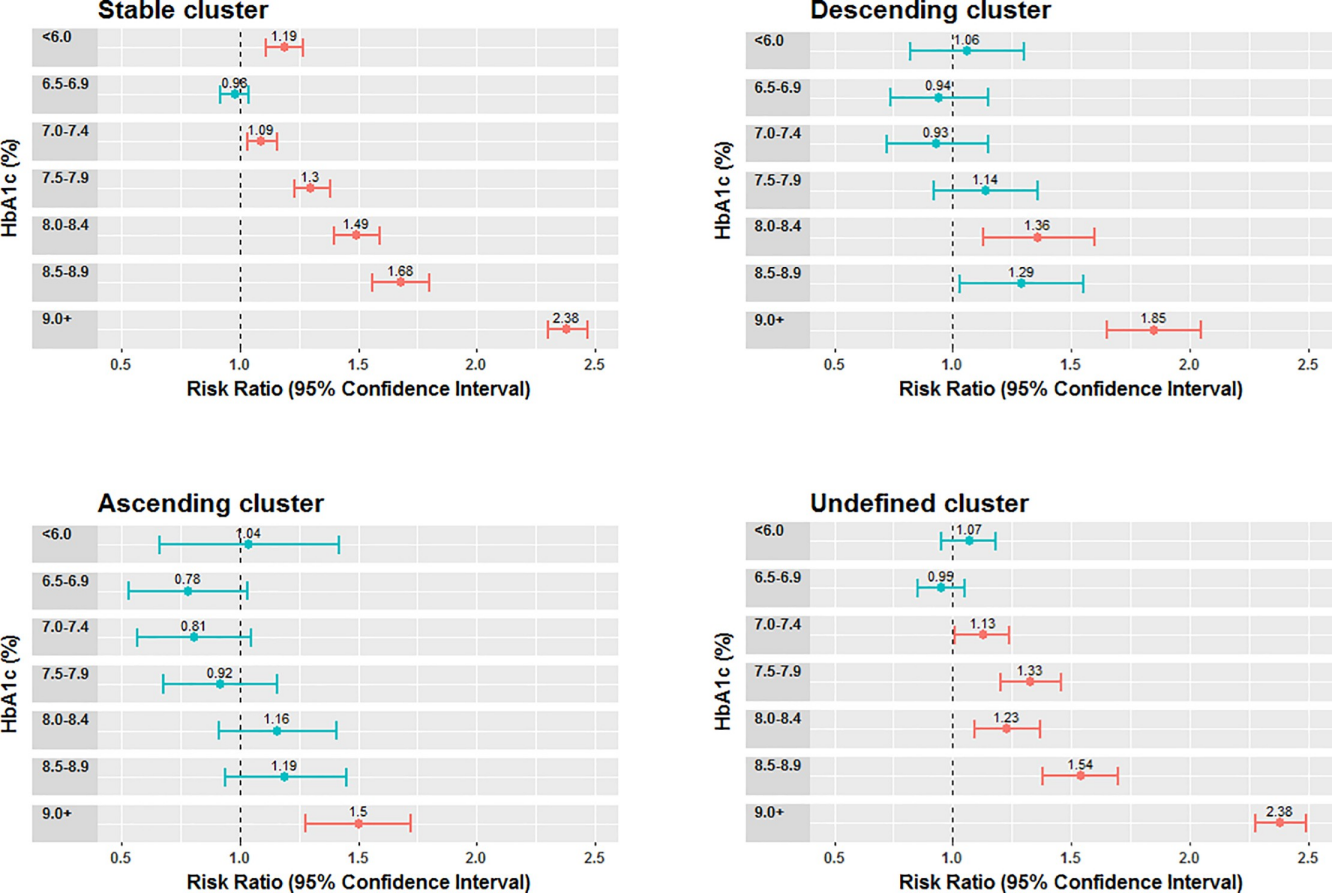
Adjusted odds ratios for the risk of having a future composite type 2 diabetes outcome. Red circles represent a significantly increase in risk (p <0.05) and black circles represent risk that is not significantly different to that of the reference group (6.0–6.4%).

Fig 3 shows the risk levels for the composite outcome by the post-index level of HbA1c after adjustment for potential confounders. There were 3,900 (4.5%) patients with a missing post-index HbA1c value who were not included in this analysis, of which 82% pertain to the NA group. A J-shape was observed among all clusters with higher risk for lower levels of HbA1c. The risk increased with the increase of HbA1c levels in all clusters. For the stable cluster, the risk was significant at an HbA1c level below 6.0% (42 mmol/mol) and for levels of 7.0% (53.0 mmol/mol) and higher. For the descending cluster risk was significant at 8.0–8.4% (64–69 mmol/mol) and at 9.0% (75 mmol/mol) and higher, and for the ascending cluster, at 9.0% (75 mmol/mol) and higher. The undefined cluster had a significantly higher risk at an HbA1c level of 7.5% (58 mmol/mol) or higher, similar to the stable cluster.

## Discussion

By stratifying a population with type 2 diabetes according to patients’ HbA1c trajectories, reproducing these clusters through supervised learning techniques, and testing these clusters for clinical relevance in terms of risk for outcomes, this study offers empirically-derived patient groups that can be used as a first step toward modeling individualized HbA1c targets. This methodology differentiates clusters of patients with distinct baseline characteristics and differential risk patterns for type 2 diabetes outcomes. The results also emphasize the importance of examining risk factors for chronic diseases like type 2 diabetes as trajectories of the course they take over time, rather than as single measurements. The reproducibility and stability of these generated clusters provides the ability to translate these clusters to other populations that have similar characteristics to the population used in this study, such as moderate diabetes disease duration.

Employing an unsupervised machine learning clustering technique offers an advantage over using population-based risk models, in which the most common characteristics influencing the vast majority of patients are identified, at the expense of potentially masking important characteristics relevant to smaller sub-groups of individuals. Such population-based models have been shown to fail when applied to some individual patients to determine individualized risk [25]. As an alternative, it has been proposed that "personalized" predictive models be built for patients based on the information of clinically similar patients [26]. Therefore, the strength of employing an unsupervised algorithm technique to determine patient clusters is that the patterns of features in the data are not predetermined, but rather, derived from what can be uncovered in the data through this methodology.

The groups of patients generated in the clustering analysis make sense clinically, as the progression of the disease may be different for those who are stable compared to those whose HbA1c level is increasing (unstable) or decreasing (responding to treatment). While international guidelines generally recommend targeting HbA1c to a value below 7.0%, except for older and the most comorbid patients, this study has identified at least two groups of patients for whom an HbA1c value associated with the lowest risk profile deviates from this recommendation. the descending and ascending clusters are classifications of patients with type 2 diabetes whose associated risk for outcomes indicates wider target HbA1c ranges. Risk of complications among the ascending cluster patients was only significant at the higher levels of HbA1c, which may signal that HbA1c is not the most important risk factor in this group of patients, thereby warranting further exploration to identify more relevant factors. The narrowest range of target HbA1c levels from 6.0–7.0%, was found for the stable cluster, in agreement with the upper bound of guideline recommendations. However, the significantly higher risk of type 2 diabetes complications associated with HbA1c <6.0% in this cluster, deviates from the guideline recommendation and is consistent with J-shaped risk curves found in previous studies [3].

There are several limitations in the study that should be taken into consideration. One limitation is that the algorithm applied in the Clalit population with type 2 diabetes requires at least one HbA1c measurement to be taken every nine months, over a period of three years, but the time between measures may vary among other populations, which should be checked. Another limitation is the large number of patients in the database who had missing HbA1c values and therefore not enough data to determine trajectories. We decided to analyze these patients separately, as the undefined cluster, and not to impute missing HbA1c values because the underlying analysis relied on the observed HbA1c value trajectories, and we did not want to introduce excessive bias based on too many imputed values. A third limitation is that this study only takes into account the five-year risk for type 2 diabetes outcomes (macrovascular, microvascular, hypoglycemic events, or all-cause mortality) among patients with relatively short duration (3–7 years) of type 2 diabetes. For some of the patients, the risk of outcomes may be higher with a longer follow-up period and thus, may generate a more comprehensive risk score. Finally, the study period was not long enough for us to study fluctuations in the trajectories of HbA1c, and with longer study periods, these clusters would likely be further refined.

Our results confirm the importance of stratifying the heterogeneous population with type 2 diabetes into more homogeneous groups through the discovery of new patterns in data. To identify relevant clusters of patients with moderate diabetes disease duration in a different context, this three-step process of conducting unsupervised learning, reproducing results in supervised learning models, and testing these clusters for clinical relevance can be replicated. This methodology can be built upon to develop more precise models that identify any individual's HbA1c target ranges with the lowest associated risk of future complications.

## Supporting information

S1 Checklist STROBE checklist of items that should be included in reports of cohort studies. (DOCX)

S1 Fig Imputation of missing HbA1c trajectory measures. (DOCX)

S1 Table Trajectory features derived from the original longitudinal HbA1c measures. (DOCX)

S2 Table Diseases definition for comorbidities and outcomes. (DOCX)

S3 Table The resulting indexes obtained by running 26 different methods available in the *“NbClust”* algorithm in R (those result are retrieved using the following code ‘nbcl$All.index’). (DOCX)

S1 Text Selection of the number of clusters. (DOCX)
