# Supplementary material for: Patient clusters based on HbA1c trajectories: A step toward individualized medicine in type 2 diabetes
Source: PLoS One. 2018 Nov 14;13(11):e0207096. doi: 10.1371/journal.pone.0207096 (PMC6235308; doi:10.1371/journal.pone.0207096)
Supplement: S1 Checklist — (DOCX) [file pone.0207096.s001.docx]

S1-Checklist. STROBE Statement—Checklist of items that should be included in reports of ***cohort studies***

|  | Item No | Recommendation | Page  No. | Relevant text from  manuscript |
| --- | --- | --- | --- | --- |
| **Title and abstract** | 1 | (*a*) Indicate the study’s design with a commonly used term in the title or the abstract | 1 | HbA1c trajectories |
|  |  | (*b*) Provide in the abstract an informative and balanced summary of what was done and what was  found | 2 | Lines 29-47 |
| Introduction | | |  |  |
| Background/rationale | 2 | Explain the scientific background and rationale for the investigation being reported | 3 | Lines 61-70 |
| Objectives | 3 | State specific objectives, including any prespecified hypotheses | 4 | Lines 71-75, hypothesis  on lines 61-62 |
| Methods | | |  |  |
| Study design | 4 | Present key elements of study design early in the paper | 4 | Lines 89-96 |
| Setting | 5 | Describe the setting, locations, and relevant dates, including periods of recruitment, exposure,  follow-up, and data collection | 4-5 | Lines 88-112 |
| Participants | 6 | (*a*) Give the eligibility criteria, and the sources and methods of selection of  participants. Describe methods of follow-up | 5 | Lines 112-113 |
|  |  | (*b*) For matched studies, give matching criteria and number of exposed and unexposed | -- | -- |
| Variables | 7 | Clearly define all outcomes, exposures, predictors, potential confounders, and effect modifiers.  Give diagnostic criteria, if applicable | 7-8  S2 | Lines 158-185  -- |
| Data sources/ measurement | 8* | For each variable of interest, give sources of data and details of methods of assessment  (measurement). Describe comparability of assessment methods if there is more than one group | S2 | -- |
| Bias | 9 | Describe any efforts to address potential sources of bias | -- | -- |
| Study size | 10 | Explain how the study size was arrived at | 6 | Lines 122-124 |
| Quantitative variables | 11 | Explain how quantitative variables were handled in the analyses. If applicable, describe which  groupings were chosen and why | -- | -- |
| Statistical methods | 12 | (*a*) Describe all statistical methods, including those used to control for confounding | 6-7  8 | Lines 131-156  Lines 186-188 |
|  |  | (*b*) Describe any methods used to examine subgroups and interactions | 6-7 | Lines 131-156 |
|  |  | (*c*) Explain how missing data were addressed | 6 | Lines 125-128 |
|  |  | (*d*) If applicable, explain how loss to follow-up was addressed | -- | -- |
|  |  | (*e*) Describe any sensitivity analyses |  |  |
| Results | | |  |  |
| Participants | 13* | (a) Report numbers of individuals at each stage of study—eg numbers potentially eligible,  examined for eligibility, confirmed eligible, included in the study, completing follow-up,  and analysed | 9 | Lines 190-191 |
|  |  | (b) Give reasons for non-participation at each stage | -- | -- |
|  |  | (c) Consider use of a flow diagram | -- | -- |
| Descriptive data | 14* | (a) Give characteristics of study participants (eg demographic, clinical, social) and information  on exposures and potential confounders | 9-12 | Table 1 |
|  |  | (b) Indicate number of participants with missing data for each variable of interest | 9-12 | Table 1 |
|  |  | (c) Summarise follow-up time (eg, average and total amount) | -- | -- |
| Outcome data | 15* | Report numbers of outcome events or summary measures over time | 9-12 | Table 1 |
| Main results | 16 | (*a*) Give unadjusted estimates and, if applicable, confounder-adjusted estimates and their  precision (eg, 95% confidence interval). Make clear which confounders were adjusted for  and why they were included | -- | -- |
|  |  | (*b*) Report category boundaries when continuous variables were categorized | 8 | Lines 181-185 |
|  |  | (*c*) If relevant, consider translating estimates of relative risk into absolute risk for a  meaningful time period | -- | -- |
| Other analyses | 17 | Report other analyses done—eg analyses of subgroups and interactions, and sensitivity  analyses | 12  13 | Lines 221-226  Lines 236-237 |
| Discussion | | |  |  |
| Key results | 18 | Summarise key results with reference to study objectives | 16 | Lines 280-283 |
| Limitations | 19 | Discuss limitations of the study, taking into account sources of potential bias or imprecision.  Discuss both direction and magnitude of any potential bias | 17 | Lines 314-327 |
| Interpretation | 20 | Give a cautious overall interpretation of results considering objectives, limitations, multiplicity  of analyses, results from similar studies, and other relevant evidence | 18 | Lines 328-331 |
| Generalisability | 21 | Discuss the generalisability (external validity) of the study results | 18 | Lines 328-331 |
| Other information | | |  |  |
| Funding | 22 | Give the source of funding and the role of the funders for the present study and, if applicable,  for the original study on which the present article is based | -- | Required by Plos to be  stated outside the manuscript |

*Give information separately for exposed and unexposed groups.

**Note:** An Explanation and Elaboration article discusses each checklist item and gives methodological background and published examples of transparent reporting. The STROBE checklist is best used in conjunction with this article (freely available on the Web sites of PLoS Medicine at http://www.plosmedicine.org/, Annals of Internal Medicine at http://www.annals.org/, and Epidemiology at http://www.epidem.com/). Information on the STROBE Initiative is available at http://www.strobe-statement.org.
