# Supplementary material for: Patient clusters based on HbA1c trajectories: A step toward individualized medicine in type 2 diabetes
Source: PLoS One. 2018 Nov 14;13(11):e0207096. doi: 10.1371/journal.pone.0207096 (PMC6235308; doi:10.1371/journal.pone.0207096)
Supplement: S1 Fig — (DOCX) [file pone.0207096.s002.docx]

# S1 Fig. Imputation of missing HbA1c trajectory measures.

The number of missing HbA1c values for the four time frames were as follows: t1: 7,136 (11.7%), t2: 5,252 (8.6%), t3: 6,390 (10.5%) and t4: 5,781 (9.5%).

The R^2^ of the models for imputation were: t1: 0.55, t2: 0.66, t3: 0.70 and t4: 0.63, based on the test dataset.


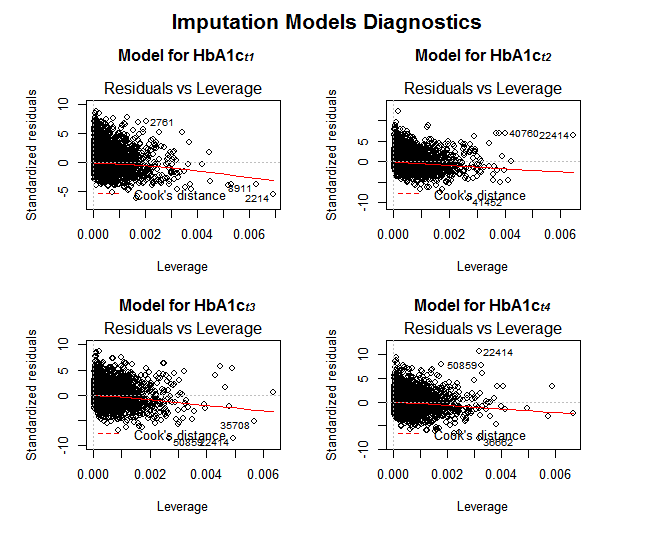


**Sensitivity analysis for missing imputation:**

From the full dataset (74,169), 30,154 had four measures (40.7%) and 23,745 (32.0%) had three measures. As we used only those with three or more measures (total 53,899), we have that those with only three measures represented 44.1%. We assessed two different sensitivity analyses for comparing the linear models we used for imputing the fourth value on those having only three values:

1. Mean between previous and following measure for those with missing in the middle and copy of the following or previous measure for those having missing in the first or last measure, respectively.
2. Mean substitution: we substitute the missing values with the mean value for each time period.

The clusters we obtain for each of the alternative imputations are very similar to that obtained for the original imputation technique. For this sub-analysis we show both the percent of similarity for the full dataset and for the imputed dataset alone.

For the first method, we got 97.8% agreement in the clusters for the full dataset and 95.9% for the imputed only data. For the second method we get 91.5% agreement in the clusters for the full dataset and 87.0% for the imputed only data. The agreement between the first and second methods was 90.6% for the full dataset and 84.5% among the imputed data. As we can see, there is a high level of agreement between the originally reported clusters and the methods used in the sub-analysis indicating that the methodology is stable.
