# Supplementary material for: Patient clusters based on HbA1c trajectories: A step toward individualized medicine in type 2 diabetes
Source: PLoS One. 2018 Nov 14;13(11):e0207096. doi: 10.1371/journal.pone.0207096 (PMC6235308; doi:10.1371/journal.pone.0207096)
Supplement: S1 Table — (DOCX) [file pone.0207096.s003.docx]

# S1 Table. Trajectory features derived from the original longitudinal HbA1c measures.

| **Features** | **Formula** |
| --- | --- |
| 1. Range | $\max(y_{i})-min(y_{i})$ |
| 2. Mean-over-time | $\bar{y}= \frac{1}{k}\sum_{i=1}^{k} y_{i}$ |
| 3. Standard deviation | $s_{y}= \sqrt{{\frac{1}{k-1}\left( y_{i}-\bar{y} \right)}^{2}}$ |
| 4. Coefficient of variation | $100 \times\frac{s_{y}}{\bar{y}}$ |
| 5. Change | $y_{k}- y_{1}$ |
| 6. Mean change per unit time | $\frac{y_{k}- y_{1}}{t_{k}- t_{1}+1}$ |
| 7. Change relative to the first score | $\frac{y_{k}- y_{1}}{y_{1}}$ |
| 8. Change relative to the mean over time | $\frac{y_{k}- y_{1}}{\bar{y}}$ |
| 9. Slope of the linear model | $b= \frac{\sum_{i=1}^{k} (y_{i}-\bar{y})(t_{i}-\bar{t})}{\sum_{i=1}^{k} {(t_{i}-\bar{t})}^{2}}$ |
| 10. R^2^: Proportion of variance explained by the linear model | $R^{2}= b^{2} \times\frac{\sum_{i=1}^{k} {(t_{i}-\bar{t})}^{2}}{\sum_{i=1}^{k} {(y_{i}-\bar{y})}^{2}}$ |
| 11. Maximum of the first differences | $max(\Delta_{1,i})$ |
| 12. Standard deviation of the first differences | $s_{\Delta_{1}}=\frac{1}{k-2}\sum_{i=1}^{k-1} \left( \Delta_{1,i}-\bar{\Delta}_{1} \right)^{2}$  where $\Delta_{1}= \frac{1}{k-1}\sum_{i=1}^{k-1} \Delta_{1,i}$ |
| 13. Standard deviation of the first differences per time unit | $s_{\Delta_{1}^{'}}=\frac{1}{k-2}\sum_{i=1}^{k-1} \left( {\Delta'}_{1,i}-{\bar{\Delta}'}_{1} \right)^{2}$  where ${\Delta^{'}}_{1}= \frac{\Delta_{1,i}}{t_{i-1}-t_{i}}$ |
| 14. Mean of the absolute first differences | $\left\vert\Delta_{1} \right\vert= \frac{1}{k-1}\sum_{i=1}^{k-1} \left\vert\Delta_{1,i} \right\vert$ |
| 15. Maximum of the absolute first differences | $max(\left\vert\Delta_{1,i} \right\vert)$ |
| 16. Ratio of the maximum absolute difference to the mean-over-time | $\frac{max(\left\vert\Delta_{1,i} \right\vert)}{\bar{y}}$ |
| 17. Ratio of the maximum absolute first difference to the slope | $\frac{max(\left\vert\Delta_{1,i} \right\vert)}{b}$ |
| 18. Ratio of the standard deviation of the first differences to the slope | $\frac{s_{\Delta_{1}}}{b}$ |
| 19. Mean of the second differences | $\bar{\Delta}_{2}= \frac{1}{k-2}\sum_{i=1}^{k-2} \Delta_{2,i}$ |
| 20. Mean of the absolute second differences | $\left\vert\Delta_{12} \right\vert= \frac{1}{k-2}\sum_{i=1}^{k-2} \left\vert\Delta_{2,i} \right\vert$ |
| 21. Maximum of the absolute second differences | $max(\left\vert\Delta_{2,i} \right\vert)$ |
| 22. Ration of the maximum absolute second difference to the mean-over-time | $\frac{max(\left\vert\Delta_{2,i} \right\vert)}{\bar{y}}$ |
| 23. Ratio of the maximum absolute second difference to mean absolute first difference | $\frac{max(\left\vert\Delta_{2,i} \right\vert)}{\left\vert\bar{\Delta}_{1} \right\vert}$ |
| 24. Ratio of the mean absolute second difference to the mean absolute first difference | $\frac{\left\vert\Delta_{2,i} \right\vert}{\left\vert\bar{\Delta}_{1} \right\vert}$ |
