# Supplementary material for: Patient clusters based on HbA1c trajectories: A step toward individualized medicine in type 2 diabetes
Source: PLoS One. 2018 Nov 14;13(11):e0207096. doi: 10.1371/journal.pone.0207096 (PMC6235308; doi:10.1371/journal.pone.0207096)
Supplement: S2 Table — (DOCX) [file pone.0207096.s004.docx]

# S2 Table. Diseases definition for comorbidities and outcomes

| **Diagnoses** | **ICD-9-CM** | **ICPC** | **Chronic World^*^** | **MoH Procedure**^†^ |
| --- | --- | --- | --- | --- |
| MI | 410; 410.0; 410.00; 410.01; 410.1; 410.10; 410.11; 410.2; 410.20; 410.21; 410.3; 410.30; 410.31; 410.4; 410.40; 410.41; 410.5; 410.50; 410.51; 410.6; 410.60; 410.61; 410.7; 410.70; 410.71; 410.8; 410.80; 410.81; 410.9; 410.90; 410.91 |  |  |  |
| UAP | 411; 411.1; 411.8; 411.81; 411.89; 413.0; 413.1 |  |  |  |
| CABG | 36.1; 36.10; 36.11; 36.12; 36.13; 36.14; 36.15; 36.16; 36.17; 36.19; 36.2; 36.3; 36.31; 36.32; 36.33; 36.34; 36.39 |  |  | G0072 |
| PTCA | 36.0; 36.01; 36.02; 36.03; 36.04; 36.05; 36.06; 36.07; 36.09 |  |  | G0085; G00T8 |
| DR | 250.5; 250.50; 250.51; 250.52; 250.53; 362.0; 362.01; 362.02; 362.03; 362.04; 362.05; 362.06; 362.07; 362.10 |  |  |  |
| DNeu | 250.6; 250.60; 250.61; 250.62; 250.63; 357.2; 536.3 |  |  |  |
| DNeph | 250.4;250.40;250.41;250.42;250.43 |  | 199.1 |  |
| LEU | 250.8; 250.80; 250.81; 250.82; 250.83; 707.1; 707.10; 707.11; 707.12; 707.13; 707.14; 707.15; 707.19; 77.68 | S97 |  |  |
| LEA | 84.1; 84.10; 84.11; 84.12; 84.13; 84.14; 84.15; 84.16; 84.17; 84.18; 84.19 | K1800 | 128.1 ;128.9 | BC302; BC325; K1800 |
| Hypoglycemic events | 251.2 | T87 |  |  |
| CVA | 430; 431; 432; 432.0; 432.1; 432.9; 433; 433.0; 433.00; 433.01; 433.1; 433.10; 433.11; 433.2; 433.20; 433.21; 433.3; 433.30; 433.31; 433.8; 433.80; 433.81; 433.9; 433.90; 433.91; 434; 434.0; 434.00; 434.01; 434.1; 434.10; 434.11; 434.9; 434.90; 434.91; 436 |  |  |  |
| Hypertension |  |  | 120.1; 120.2 ;120.9 |  |

Abbreviations: ICD-9-CM, international classification of diseases, ninth revision, clinical modification; ICPC, international classification of primary care; MoH, ministry of health; MI, myocardial infarction; UAP, unstable angina pectoris; CABG, coronary artery bypass graft; PTCA, percutaneous transluminal coronary angioplasty; DR, diabetic retinopathy; DNeu, diabetic neuropathy; DNeph, diabetic nephropathy; LEU, low extremity ulcers; LEA, low extremity amputations; CVA, cerebrovascular disease.

^*^Diseases based on the Clalit’s internal definition of chronic diseases.

^†^Procedures defined by the Israeli Ministry of health. Codes are based on the CPT classification.
