# Supplementary material for: Patient clusters based on HbA1c trajectories: A step toward individualized medicine in type 2 diabetes
Source: PLoS One. 2018 Nov 14;13(11):e0207096. doi: 10.1371/journal.pone.0207096 (PMC6235308; doi:10.1371/journal.pone.0207096)
Supplement: S3 Table — (DOCX) [file pone.0207096.s005.docx]

# S3 Table. The resulting indexes obtained by running 26 different methods available in the *“NbClust”* algorithm in R (those result are retrieved using the following code ‘nbcl$All.index’)

| **Method** | **Number of clusters** | | | | | | |
| --- | --- | --- | --- | --- | --- | --- | --- |
|  | **2** | **3** | **4** | **5** | **6** | **7** | **8** |
| KL | 1.074 | 3.0665 | 1.5227 | 2.1851 | 0.1858 | 1.9503 | 2.0589 |
| CH | 8212.555 | 9765.8 | 9632.788 | 9390.806 | 8842.189 | 9087.798 | 9064.839 |
| Hartigan | 7815.698 | 4533.595 | 3361.955 | 2179.529 | 3022.223 | 2245.09 | 1758.792 |
| CCC | 128.6304 | 125.8467 | 108.8517 | 101.1549 | 93.2224 | 98.312 | 123.1428 |
| Scott | 55458.96 | 71121.76 | 81976.18 | 91890.56 | 99249.81 | 106245.33 | 111248.11 |
| Marriot | 1.38E+12 | 1.32E+12 | 1.30E+12 | 1.18E+12 | 1.14E+12 | 1.06E+12 | 1.05E+12 |
| TrCovW | 82590748 | 33751580 | 17783033 | 14952786 | 12509712 | 9140328 | 6630349 |
| TraceW | 24428.428 | 17122.159 | 13724.798 | 11596.072 | 10362.746 | 8894.751 | 7923.286 |
| Friedman | 12.0475 | 13.5961 | 17.8635 | 25.1176 | 26.5312 | 28.7814 | 30.8713 |
| Rubin | 3.5552 | 5.0723 | 6.3279 | 7.4895 | 8.3809 | 9.7641 | 10.9612 |
| Cindex | 0.1268 | 0.1141 | 0.1039 | 0.099 | 0.1015 | 0.0973 | 0.1007 |
| DB | 1.4527 | 1.0722 | 1.1646 | 1.1355 | 1.0324 | 0.9915 | 1.0242 |
| Silhouette | 0.3107 | 0.4319 | 0.3006 | 0.3127 | 0.3223 | 0.3229 | 0.312 |
| Duda | 0.8299 | 0.9454 | 0.9422 | 2.0142 | 1.1095 | 1.3864 | 1.5354 |
| Pseudot2 | 3193.0311 | 609.3094 | 351.879 | -3695.4187 | -655.1801 | -2444.3173 | -2388.3255 |
| Beale | 0.349 | 0.0983 | 0.1044 | -0.857 | -0.1681 | -0.4744 | -0.5935 |
| Ratkowsky | 0.2688 | 0.3156 | 0.3321 | 0.3307 | 0.3215 | 0.3059 | 0.2904 |
| Ball | 12214.2139 | 5707.3863 | 3431.1994 | 2319.2145 | 1727.1243 | 1270.6787 | 990.4108 |
| Ptbiserial | 0.2781 | 0.6069 | 0.4622 | 0.4087 | 0.4199 | 0.4277 | 0.4078 |
| Frey | -1.859 | 4.3623 | 1.3477 | 0.0549 | 0.16 | 1.0643 | 1.1544 |
| McClain | 0.5953 | 0.2851 | 0.7381 | 1.19 | 1.2047 | 1.2423 | 1.4272 |
| Dunn | 0.0004 | 0.0012 | 0.0006 | 0.0003 | 0.0009 | 0.0009 | 0.0008 |
| Hubert | 0.00E+00 | 1.00E-04 | 1.00E-04 | 1.00E-04 | 1.00E-04 | 1.00E-04 | 1.00E-04 |
| SDindex | 3.4625 | 3.6862 | 4.5252 | 4.5391 | 4.4861 | 4.7213 | 5.0377 |
| Dindex | 0.9261 | 0.8216 | 0.7281 | 0.6565 | 0.6307 | 0.5921 | 0.5615 |
| SDbw | 1.9902 | 0.8941 | 0.8913 | 0.8293 | 0.7292 | 0.6024 | 0.5875 |
