# Supplementary material for: Patient clusters based on HbA1c trajectories: A step toward individualized medicine in type 2 diabetes
Source: PLoS One. 2018 Nov 14;13(11):e0207096. doi: 10.1371/journal.pone.0207096 (PMC6235308; doi:10.1371/journal.pone.0207096)
Supplement: S1 Text — (DOCX) [file pone.0207096.s006.docx]

# S1 Text. Selection of the number of clusters

| > nbcl <- NbClust(data,method="kmeans",min.nc=2,max.nc=8,index="all")  ***: The Hubert index is a graphical method of determining the number of clusters.  In the plot of Hubert index, we seek a significant knee that corresponds to a  significant increase of the value of the measure i.e the significant peak in Hubert index second differences plot.  ***: The D index is a graphical method of determining the number of clusters.  In the plot of D index, we seek a significant knee (the significant peak in Dindex second differences plot) that corresponds to a significant increase of the value of the measure.    *******************************************************************  * Among all indices:  * 5 proposed 2 as the best number of clusters  * **11 proposed 3 as the best number of clusters**  * 1 proposed 4 as the best number of clusters  * 3 proposed 5 as the best number of clusters  * 2 proposed 7 as the best number of clusters  * 1 proposed 8 as the best number of clusters  ***** Conclusion *****  * According to the majority rule, the best number of clusters is 3  ******************************************************************* |
| --- |
